# Congenital Methemoglobinemia-Induced Cyanosis in Assault Victim

**DOI:** 10.7759/cureus.14079

**Published:** 2021-03-24

**Authors:** Atheer T Alotaibi, Abdullah A Alhowaish, Abdullah Alshahrani, Dunya Alfaraj

**Affiliations:** 1 Medicine Department, Imam Abdulrahman Bin Faisal University, Dammam, SAU; 2 Emergency Department, Imam Abdulrahman Bin Faisal University, Dammam, SAU

**Keywords:** methemoglobinemia, cyanosis, hypoxia, trauma

## Abstract

Methemoglobinemia is a blood disorder in which there is an elevated level of methemoglobin. In contrast to normal hemoglobin, methemoglobin does not bind to oxygen, which leads to functional anemia. The signs of methemoglobinemia often overlap with other cardiovascular and pulmonary diseases, with cyanosis being the key sign of methemoglobinemia. Emergency physicians may find it challenging to diagnose cyanosis as a result of methemoglobinemia.

Our patient is a healthy 28-year-old male, a heavy smoker, who presented to the emergency department with multiple minimum bruises on his body, claiming he was assaulted at work. He appeared cyanotic with an O_2_ saturation of 82% (normal range is 95-100%) in room air. He also mentioned that his sister complained of a similar presentation of cyanosis but was asymptomatic. All these crucial points strengthened the idea that methemoglobinemia was congenital in this patient.

The case was challenging to the emergency physician, and there was significant controversy over whether the patient's hypoxia was a result of the trauma or congenital methemoglobinemia.

## Introduction

Methemoglobinemia is an important cause of cyanosis; however, clinical cyanosis is challenging in regard to forming a concrete diagnosis as causes are multiple especially in the absence of cardiopulmonary causes [1]. Methemoglobin is an abnormal form of hemoglobin. The core function of hemoglobin is to carry oxygen in the red blood cells; however, if the ferrous form of hemoglobin (Fe2+) becomes oxidized, it will convert to its ferric (Fe3+) form which will cause the RBC (red blood cell) to lose its ability to carry oxygen, thus forming methemoglobin. Methemoglobinemia is an elevated level of methemoglobin in the blood leading to cyanosis and hypoxia due to the decreased oxygen supply to that tissue. Unfortunately, this does not improve with oxygen therapy [1].

Methemoglobinemia is suspected in the presence of dyspnea or unexplained cyanosis and hypoxemia that does not improve with supplemental oxygen. The severity of the presentation may vary from asymptomatic cyanosis in mild cases to severe distress. Usually, the severity of the symptoms correlates with the methemoglobin level. The susceptibility of the diagnosis will increase if there is positive family history, previous history of methemoglobinemia, or exposure to a substance that is known to induce methemoglobinemia [1].

Methemoglobinemia is mainly diagnosed clinically and is confirmed by direct measurement of methemoglobinemia by arterial or venous blood gas with co-oximetry. A methemoglobin concentration of >3% (normal range is 0-3%) is the definitive diagnosis. In an emergency room (ER) setting, consider obtaining the following: finger-stick glucose, lactate, pregnancy test because methylene blue is a teratogen, CBC (complete blood count), LFT (liver function test), RFT (renal function test), and troponin to detect if there is end-organ damage due to hypoxemia. Regarding treatment, as soon as methemoglobinemia is identified, we initiate supportive care, ensure airway patent, supplemental O_2_ and hemodynamic stabilization, as well as identifying the causative agent if it is acquired [2].

Here, we would like to present a case of undiagnosed methemoglobinemia presented as a case of assault with continuous cyanosis and hypoxemia, which lead to a false initial diagnosis.

## Case presentation

Our patient was a healthy 28-year-old male who presented to the emergency department with trauma to the neck, right shoulder and the upper part of his right arm. He appeared to be in pain as a facial grimace was noticeable. He was assaulted by two people at work; however, he claimed to have no recollection of events following the initial altercation due to him losing consciousness shortly after. He did not complain of shortness of breath, nausea, or vomiting.

Due to the patient appearing cyanotic and hypoxic, he was put on a resuscitation bed for fear of severe trauma. A primary survey (ABC; Airway, Breathing, Circulation) was done and we found the airway was patent. Full backboard and rigid cervical collar were applied. Breathing: O_2_ saturation 82% in room air. The patient was then hooked to a non-breathing mask at 15 LPM (liters per minute) saturating 86% to 88%. The patient had equal bilateral air entry and symmetrical chest expansion and a respiratory rate of 22 BPM (breaths per minute; the normal rate is anywhere from 12 to 20, anything over that is considered tachypnea). He was also hooked to a cardiac monitor. Pulse was 74 bpm, regular; 20 gauge intravenous (IV) cannula was inserted in the right metacarpal vein and 18 gauge IV cannula was inserted in right brachial vein connected to hemlock. Blood was extracted and sent to the laboratory. Glasgow coma scale (GCS) was 15 (a score of 15 means the patient is completely conscious).

In a secondary survey on examination, abrasion was noted on the right forearm. Focused Assessment with Sonography for Trauma (FAST) was negative; portable chest and pelvic X-ray were clear. ER physician advised the patient to undergo CT to assess trauma, which was not possible as the patient went against the advice and left. Arterial blood gas results arrived a bit later, and showed the following: pH 7.395 (7.35-7.45), PCO_2_ (partial pressure of carbon dioxide) 42 mmHg (35-45 mmHg), low PO_2_ (partial pressure of oxygen) at 77.4 mmHg (83-108), HCO_3_ (bicarbonate) 24.8 (23-29 mmol/L), and low oxyhemoglobin at 63.8% (94-98%), high MetHb (methemoglobin) at 32% (0-1.5%). CBC, LFT, RFT and finger-stick glucose were unremarkable.

**Figure 1:**
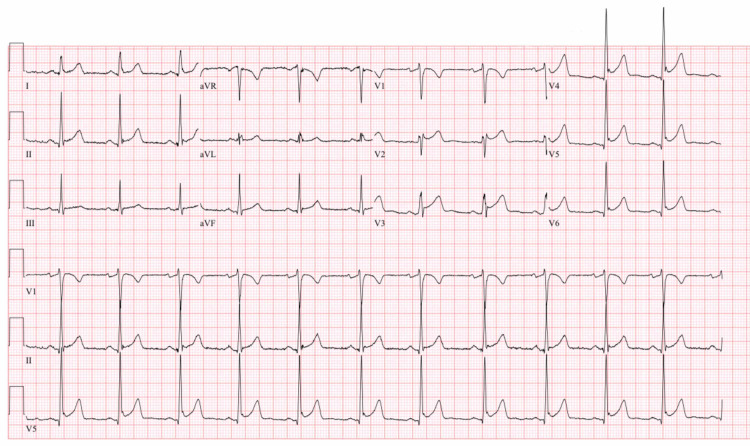
ECG (Electrocardiography)

Cardiac enzyme was positive, troponin I 0.061 ng/ml (normal range is 0-0.04 ng/ml), CPK 823 U/L (creatine phosphokinase; normal range is 39-308 U/L). Electrocardiography (ECG) showed benign early depolarization (Figure 1); however, in the context of methemoglobinemia and elevated troponin, we consulted cardiology early on to rule out STEMI (ST-segment elevated myocardial infarction). He was given 70 mg of methylene blue IV. After administration of methylene blue, his methemoglobin decreased to 1.5%. The patient was discharged via the cardiology department after his troponin went down to 0.033 ng/ml. The patient refused medication and signed discharge against medical advice.

## Discussion

Methemoglobinemia can be either congenital or acquired. The most common cause of congenital methemoglobinemia is a result of deficient cytochrome b5 reductase (CYB5R). CYB5R deficiency is a result of variations in the cytochrome b5 reductase 3 gene (CYB5R3). The variations lead to type 1 and type 2 CYB5R3. Type 1 focuses on the CYB5R deficiency in RBCs. Patients are typically asymptomatic aside from appearing cyanotic. The life expectancy of this patient would be that of the general population; however, the life expectancy of type 2 patients is extremely diminished and usually pass away at a very young age. The reason for the decreased life expectancy in type 2 is because there is CYP5R deficiency in all cells, which leads to neurological complications, microcephaly, mental retardation, etc. [3].

Hemoglobin M is a congenital cause of methemoglobinemia but is rare in comparison to CYB5R3. Persistent neonatal cyanosis is caused by variation in alpha chains. Cyanosis several months post-partum is due to variations in beta chains after a decrease in fetal hemoglobin levels. Transient neonatal cyanosis is a result of variations in gamma chain variations and is resolved when fetal hemoglobin level decreases.

Some patients with congenital methemoglobinemia will develop physiological adaptations and as result will be asymptomatic even with an elevated level of methemoglobin (up to 40%) due to changes in the concentration of 2,3-Diphosphoglycerate and pH, synthesis of globin chains and polycythemia [4].

Congenital methemoglobinemia is usually missed clinically. It is a rare, underreported hemoglobin disease. Oftentimes congenital methemoglobinemia presents in infancy in contrast to acquired methemoglobinemia which presents in older age groups. Most cases of methemoglobinemia are acquired. Acquired methemoglobinemia is the product of exposure to substance(s) (oxidizing agents) that induce the formation of methemoglobin such as benzocaine, nitrates, prilocaine, aniline, dapsone, and some antimalarial drugs such as chloroquine and primaquine [5].

A retrospective study of 138 cases of acquired methemoglobinemia for 28 months found that dapsone was the most common cause of acquired methemoglobinemia in 42%, benzocaine in 4%, and primaquine in 4% of the patients. Dapsone is an antibiotic commonly used in the treatment of leprosy; benzocaine used mainly as topical pain reliever; and primaquine is used for treating malaria [6]. In our case, the patient denied any history of drug use or environmental exposure to agents that may trigger methemoglobinemia.

The first clinical sign when methemoglobin levels reach ≥ 10% is cyanosis, but symptoms of hypoxemia and diminished oxygen transport do not appear until levels increase to 30% to 40% [7]. In our patient, the initial presentation was cyanosis due to methemoglobinemia which was initially misdiagnosed as cyanosis due to trauma. There is another reported case where methemoglobinemia was misdiagnosed as polycythemia vera and was treated with imatinib. Cyanosis due to methemoglobinemia was then suspected in our patient because of the presence of finger clubbing, O_2_ saturation of 82% on room air and no improvement after supplemental O_2_ was administered. The patient mentioned that his sister complained of a similar presentation of cyanosis. Diagnosis of methemoglobinemia was made after blood gas was reported and early treatment of methemoglobinemia was initiated but the patient refused, unlike in the other reported case of false diagnosis of polycythemia vera which led to unnecessary bone marrow examination and receiving imatinib [8].

The presence of a saturation gap can be one possible hint to the diagnosis of methemoglobinemia. A positive saturation gap is a difference of >5% between oxygen saturation measured by means of pulse oximetry and arterial blood gas analysis [8]. The earlier the diagnosis is made the better outcomes will be, especially with those who present to the emergency department as a case of trauma.

In our case, the patient presented as a case of assault with continued cyanosis and hypoxemia which lead to a false initial diagnosis of cyanosis and hypoxemia due to trauma. Usually, congenital methemoglobinemia is underdiagnosed due to the lack of systematic epidemiological studies [8]. An important point that should be taken into consideration regarding this case is that methemoglobinemia should be considered in every patient present to ER complaining of cyanosis or hypoxia, and not to focus on trauma and forget other possible underlying causes of hypoxia and cyanosis.

## Conclusions

In conclusion, methemoglobinemia should be considered in patients presenting to the ER with cyanosis following trauma or assault. Congenital methemoglobinemia is a rare disease but can be managed easily, it is one of the causes of cyanosis that should be ruled out in the setting of cyanosis without cardiorespiratory causes or exposure to oxidant.
